# Genome-wide association mapping of *Sclerotinia sclerotiorum* resistance in soybean using whole-genome resequencing data

**DOI:** 10.1186/s12870-020-02401-8

**Published:** 2020-05-07

**Authors:** Chiheb Boudhrioua, Maxime Bastien, Davoud Torkamaneh, François Belzile

**Affiliations:** Département de phytologie and Institut de Biologie Intégrative et des Systèmes (IBIS), Université Laval, Quebec City, Quebec, G1V0A6 Canada

**Keywords:** WGS, *Sclerotinia sclerotiorum*, Soybean, GWAM

## Abstract

**Background:**

Sclerotinia stem rot (SSR), caused by *Sclerotinia sclerotiorum* (Lib.) de Bary, is an important cause of yield loss in soybean. Although many papers have reported different loci contributing to partial resistance, few of these were proved to reproduce the same phenotypic impact in different populations.

**Results:**

In this study, we identified a major quantitative trait loci (QTL) associated with resistance to SSR progression on the main stem by using a genome-wide association mapping (GWAM). A population of 127 soybean accessions was genotyped with 1.5 M SNPs derived from genotyping-by-sequencing (GBS) and whole-genome sequencing (WGS) ensuring an extensive genome coverage and phenotyped for SSR resistance. SNP-trait association led to discovery of a new QTL on chromosome 1 (Chr01) where resistant lines had shorter lesions on the stem by 29 mm. A single gene (Glyma.01 g048000) resided in the same LD block as the peak SNP, but it is of unknown function. The impact of this QTL was even more significant in the descendants of a cross between two lines carrying contrasted alleles for Chr01. Individuals carrying the resistance allele developed lesions almost 50% shorter than those bearing the sensitivity allele.

**Conclusion:**

These results suggest that the new region on chromosome 1 harbors a promising resistance QTL to SSR that can be used in soybean breeding program.

## Background

Sclerotinia stem rot (SSR) is a significant disease that causes yield and quality loss in soybean in the northern United States and Canada. This disease is caused by *Sclerotinia sclerotiorum*, a necrotrophic Ascomycota, capable of infecting more than 408 different species [6]. The fungus infects the plant via the flower then spreads through the stem causing bleaching, severe wilting and shredding of tissue [7]. SSR was reported as the second most important disease-causing yield losses in Canada in 1994 and in the USA in 1994, 2004 and 2009 [18, 32]. However, the impact of this disease is very unpredictable from year to another because fungal development is highly influenced by temperature and humidity [20]. Its impact could be reduced by using chemical or biological control, but results can be variable as these methods can fail when disease incidence is higher than 50% [33]. The best results can be achieved when several preventive treatments are applied each year even when SSR doesn’t pose a threat due to unfavorable climate conditions. Considering these facts, enhancing the genetic resistance of soybean cultivars seems to be the most effective solution to reduce the detrimental impacts of SSR.

The evaluation of SSR resistance is quite challenging in variable environmental conditions. However, a reliable inoculation method was developed by Bastien et al. [3] wherein a mycelium suspension is applied on flower buds in controlled greenhouse conditions. It has been shown to produce consistent results and was used to investigate the genetic determinants of SSR resistance is soybean [4, 13, 15].

To date, complete resistance has yet to be reported in soybean. Partial resistance is controlled by multiple genes or quantitative trait loci (QTL). Numerous mapping studies have been conducted and have identified more than 114 QTL via conventional biparental mapping [1, 11, 13, 16, 17, 29, 34]. Although this method has been widely used for QTL mapping, it is still limited to the genetic diversity present in the two parents. More recently, with the advancement of genotyping technologies, it was possible to screen quantitative partial resistance in multiple soybean lines with thousands of markers using GWAM. Using this method, more than 130 QTLs have also been reported in different populations [4, 15, 21, 30, 31, 34]. Such number of loci raise some questions about their credibility especially when fewer of these were proved to reproduce the same allelic effect in different genetic backgrounds. One explanation is that some of these QTLs identified based on different methods of evaluation, could be confused with an escape or avoidance mechanisms and not genuinely related to the real physiological resistance to SSR [4, 17]. As a proof, the only QTL proved to reproduce the same phenotyping effect in a biparental cross was identified on chromosome 15 based on resistance evaluation under a controlled environment [4]. These results suggest that a reliable phenotyping method is a key factor in this study.

Compared to biparental mapping, diversity panels offer a lower level of linkage disequilibrium (LD) between markers and QTLs. Hence, for GWAM, a higher marker density is needed depending on population size and diversity. For higher QTL detection power, the LD between the QTL and any flanking markers should be higher than 0.8. To achieve such a coverage, Bastien et al. [4] estimated that at least 12,900 SNPs in the pericentromeric regions and 55,700 SNPs in the telomeric region would be needed for a total of over 68 K well-distributed SNPs to cover the entire genome. For mapping SSR resistance loci in soybean, many attempts were made to achieve such converge using different genotyping approaches like genotyping by sequencing (GBS) [4, 15, 30] or specific locus amplified fragment sequencing (SLAF-seq) [34]. To date, the largest number of informative SNPs was achieved using the SoySNP50K array in two studies. One obtained 35,683 SNPs on 466 accessions [21] and the other achieved 31,600 and 35,708 SNPs, respectively, in populations of 915 improved lines and 405 soybean landraces [31]. It is likely that the marker coverage obtained in these most recent papers still falls short of the number needed to ensure exhaustive genome coverage.

One alternative to the previously used genotyping approaches is whole-genome sequencing (WGS). However, this approach is still expensive, especially when using large populations. In previous work, Torkamaneh et al. [28] proposed a two-step approach termed “scanning and filling”. In a first step, a large population can be genotyped at tens of thousands of SNP loci (using GBS or an array). In a second step, WGS can be performed on a subset of these lines (e.g. 20%) and these can serve as a reference panel to impute millions of SNP markers onto the entire set of accessions.

In this work, we used such a combined GBS and WGS genotyping approach to genotype an association panel (comprising elite Canadian soybean lines) at millions of SNPs. We then used this exhaustive marker dataset to perform GWAM in the association panel to identify QTLs responsible for partial resistance to SSR in Canadian soybean.

## Results

### SSR resistance in lines of the association panel

**Fig. 1:**
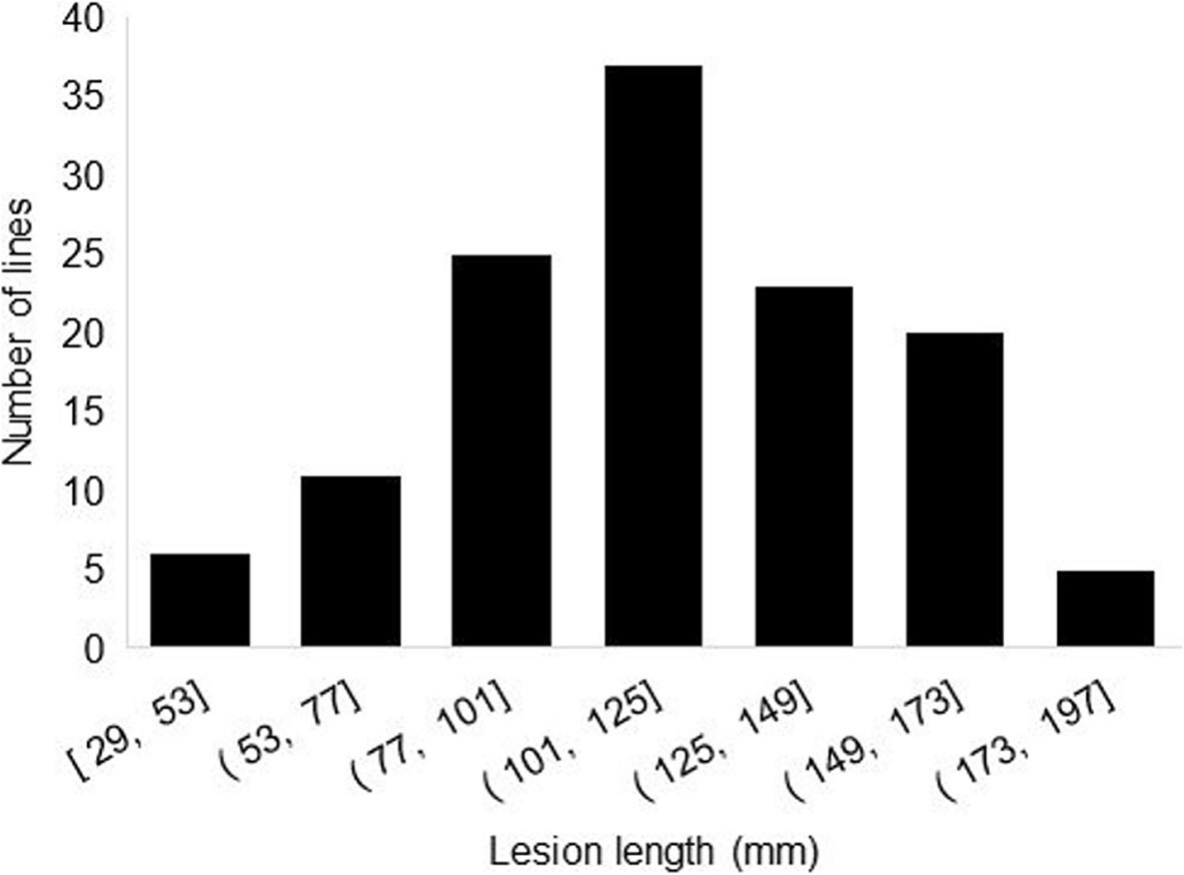
Distribution of mean lesion length observed seven days after inoculation among 127 soybean lines. [A, B]: A ≤ lesion length ≤ B. (A, B]: A < lesion length ≤ B

Lesion length was measured 7 days after inoculation on young flower buds, and the mean value for each genotype is shown in Supplementary Table S1. As illustrated in Fig. 1, lesion lengths were found to range broadly, from as low as 29 mm to a maximum of 192 mm, with lesion length in the population averaging 114 mm. The distribution of lesion lengths was bell-shaped suggesting that several genes control this trait. The resistant checks (Karlo RR, S19–90 and Maple Donovan) ranked among the lines with the shortest lesions (1st, 3rd and 21st out of 127) while the highly susceptible check Nattosan had the second longest lesions (177 mm) and the two moderately susceptible checks (Williams 82 and OAC Bayfield) showed lesions slightly above the population average.

### Marker distribution

To achieve extensive genome coverage, we re-analyzed previously obtained sequence data (940 M single-end reads from *Ape*KI GBS libraries prepared from DNA of 530 elite Canadian soybean lines) using an improved SNP-calling pipeline (Fast-GBS) and a more recent version of the soybean reference genome. This yielded nearly 150 K SNPs on the panel of 530 lines that included all lines of the association panel. We then used a catalog of 4.1 M SNPs obtained from WGS of 102 lines, also included in the set of 530 lines, as a reference panel to impute genotypes at all the missing loci, thus resulting in a full dataset of 4.1 M markers. Of these, 3.5 M SNPs were polymorphic in our association panel (i.e., carried an alternate allele in at least for one of the 127 lines). After removal of SNPs mapping to scaffolds (49.7 K SNPs), 3.4 M SNPs mapped onto one of the 20 soybean chromosomes. Finally, we removed markers with MAF lower than 0.05, thus resulting in a final catalog of 1,493,960 SNPs with which we performed the GWAM analysis.

### Population structure and kinship

**Fig. 2:**
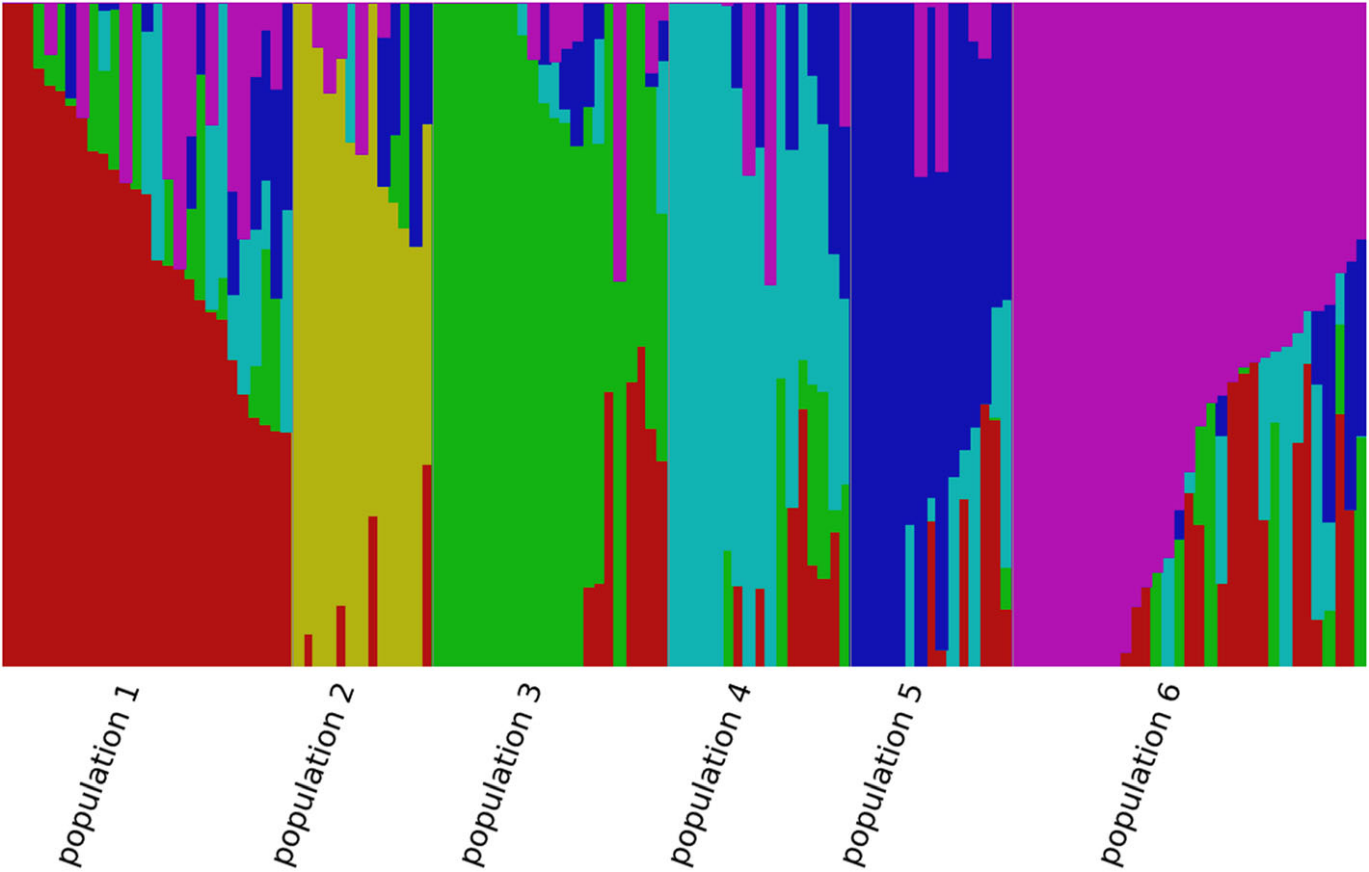
Structure plot for the 127 Canadian soybean

To characterize population structure, we pruned SNPs in high LD (r^2^ ≥ 0.9; windows of 50 SNPs), and the remaining 84,708 SNPs were used in fastSTRUCTURE. The results suggested that the panel was composed of between three and six subpopulations. Based on these two results, we chose to perform the ensuing analysis using K = 6 and the corresponding plot is shown in Fig. 2. To further reduce confounding, we estimated the kinship matrix between lines of the association panel.

### Genome-wide association mapping for SSR resistance

**Fig. 3:**
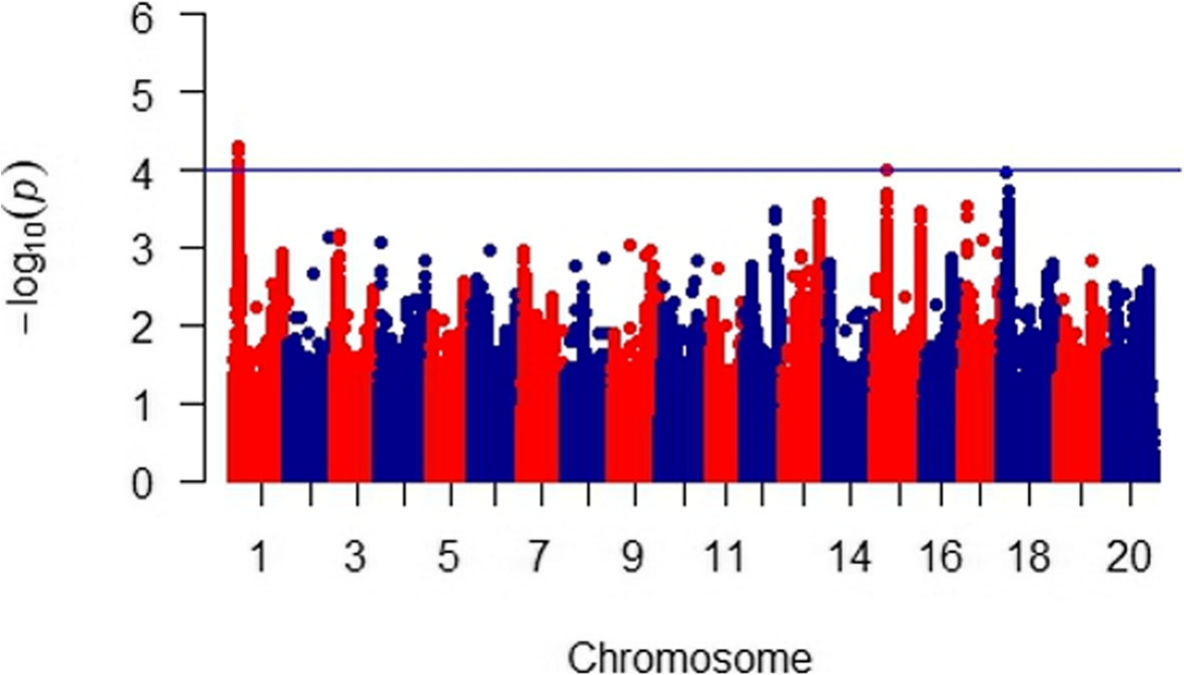
Manhattan plot of genome-wide association scan for Sclerotinia stem rot (SSR) resistance in soybean. The horizontal line indicates the significance threshold (FDR = 0.1). Peak SNP (chromosome 1:5594597; T/C)

**Fig. 4:**
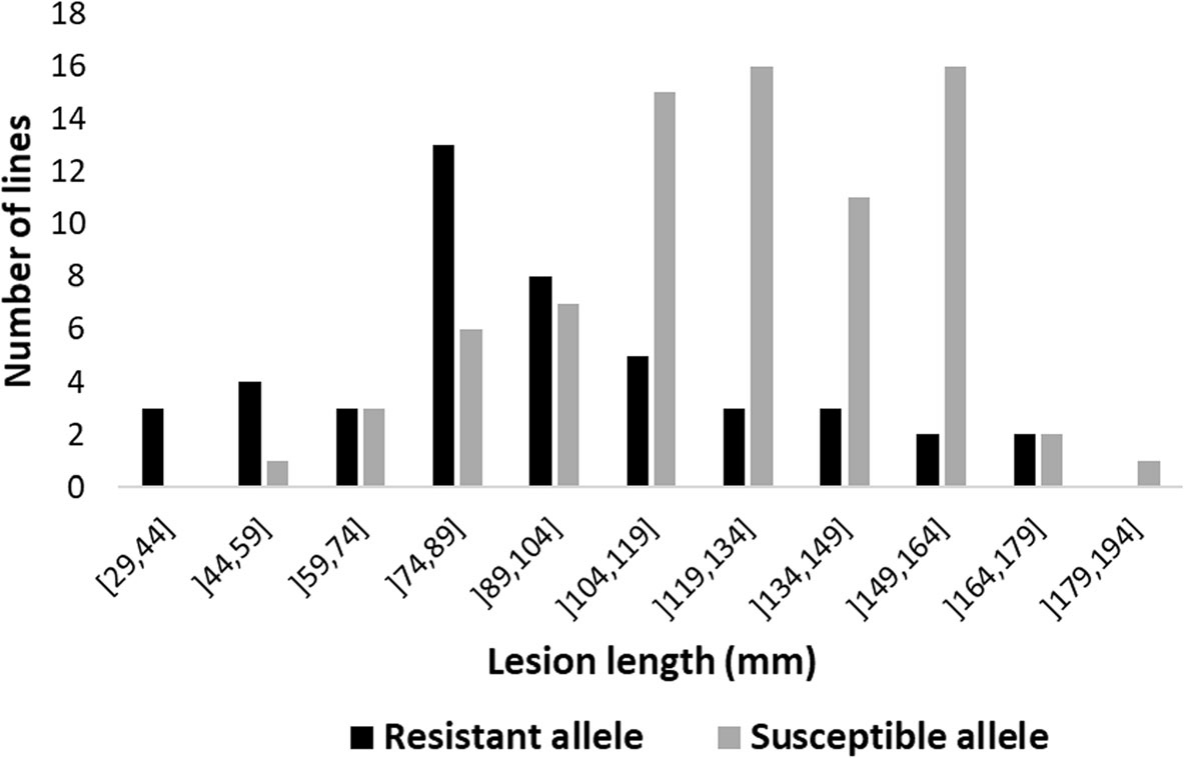
Lesion length distribution across the 127 lines according to alleles at the peak marker on Chr01

Marker-trait associations were estimated using the phenotypic data (mean lesion length) and the full set of filtered SNP markers (close to 1.5 M markers). These were analyzed using an MLM (Q + K) and associations with *p*-values corresponding to an FDR < 0.1 were considered significant. In total, only two chromosomal regions were found to have at least one peak SNP exceeding this threshold (on Chr01 Chr15; Fig. 3). As detailed in Table 1, the peak SNP on Chr01 was at position 5,594,597, showed a *p*-value of 5.08 × 10^− 5^ and explained 32% of the phenotypic variation. As shown in Fig. 4, accessions carrying the favorable allele T (frequency = 0.38) at this locus showed shorter lesions compared to those with succeptible allele C. Although some accessions still exhibited lesions averaging over 100 mm despite carriyng the resistance allele on Chr01. A second associated region was found on chromosome 15 (chr15) with a single significantly associated marker at position 13,665,369 (*p*-value = 9.76 × 10^− 5^; FDR = 0.04) and explained 15% of the variation. Accessions fixed for the minor allele A (frequency = 0.32) had lesions that were 15 mm shorter than those fixed for the major allele G (Supplementary Figure 1).

**Table 1:** Characteristics of the markers most highly associated (peak SNPs) with lesion length

Chromosome (Chr)	Position	p-value	Resistant allele/susceptible allele	FDR	MAF	R^2^	Allelic effect (mm)
01	5,594,597	5.08 × 10^−5^	T/C	0.02	0.38	0.32	29
15	13,665,369	9.76 × 10^−5^	A/G	0.04	0.32	0.15	15

Position: The physical position of the marker on the chromosome according to the *G. max* reference genome [Gmax_275 (Wm82.a2.v1)] [24]

*FDR* False discovery rate

*MAF* Minor allele frequency

R^2^: Indicates the proportion of total phenotypic variation accounted for by the marker

Allelic effect: average change in lesion length following allele substitution

### Validation experiment

**Fig. 5:**
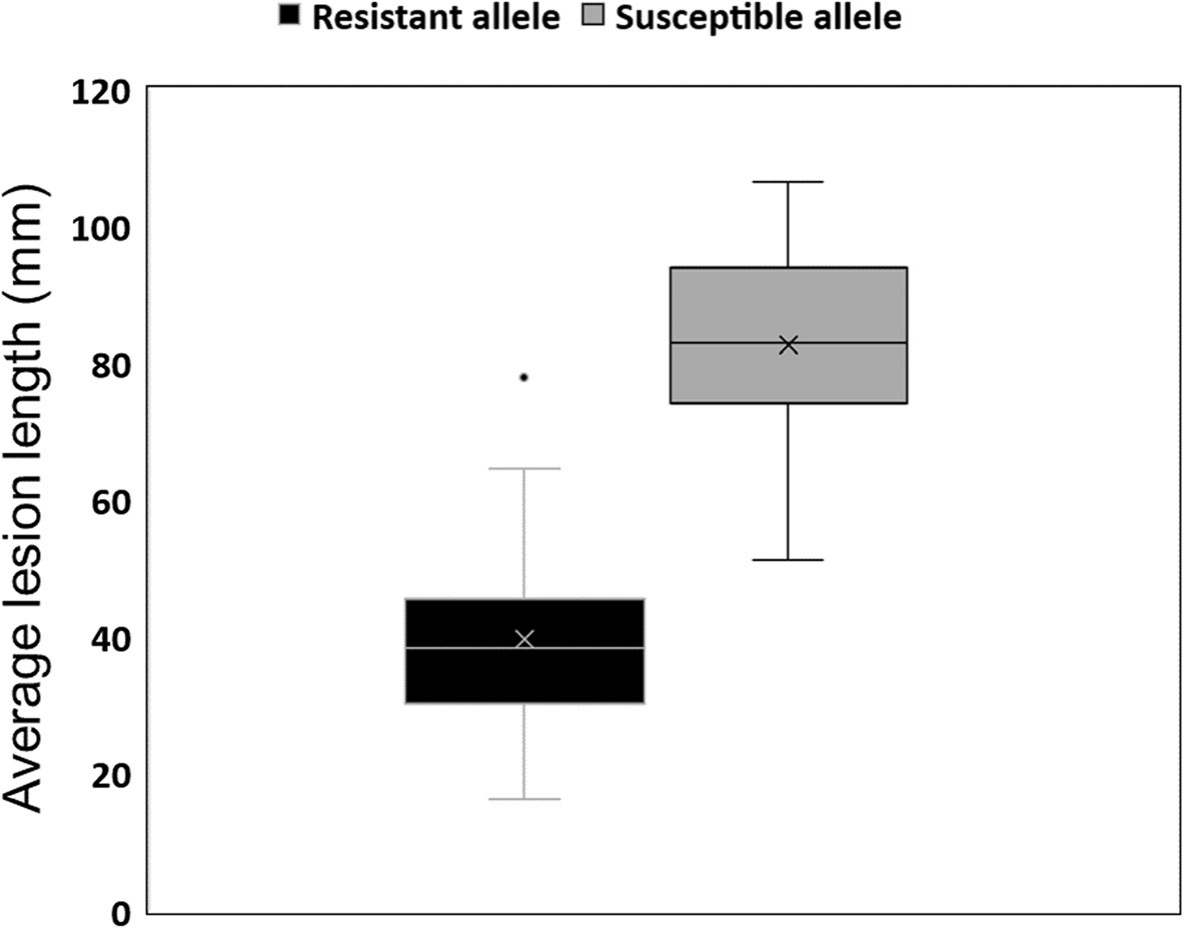
Lesion length distribution among RILs according to the fixed allele at the peak marker on Chr01

As the association on Chr15 had already been validated in previous work [4], we focused here on validating the candidate region for SSR resistance on Chr01. To do this, we used a population of F_6:8_ lines derived from a cross between OAC Bayfield (S) and Maple Donovan (R). These parents were contrasted for the peak marker on Chr01 as well for SSR resistance; Maple Donovan carries the resistance allele and developed lesions 78.3 mm shorter than those exhibited by OAC Bayfield. The parents were used as checks in the validation trial in addition to 47 recombinant inbred lines (RILs) selected as a validation population. For each line, four plants were genotyped using a CAPS marker developed to assay the QTL on Chr01. Among the 47 RILs, 21 were homozygous for the resistance allele while 26 were fixed for the susceptible allele. These RILs, along with the parents, were then evaluated for SSR resistance in two greenhouse trials. The contrast in lesion length between the parents was still evident (60 mm). Among the RILs, the average lesion length was 63 mm and ranged from 16 to 107 mm. Interestingly, almost all genotypes fixed for the resistance allele developed lesions under the average, ranging between 16 and 78 mm (average of 40 mm), whereas lines homozygous for the susceptible allele averaged 83 mm with lesion length extending from 51 to 107 mm. The phenotypic contrast between the two genotypic classes (43 mm) (Fig. 5) was significant (*p* = 0.007).

### The genomic landscape around the QTL on Chr01

The LD was estimated between all marker pairs between 5.4 and 5.8 Mb to investigate the genomic landscape in the associated region on chromosome 1. When defining LD blocks on the basis of almost perfect LD (r^2^ ≤ 0.98), all of the markers showing a significant association with lesion length resided in a small block (10 kb; block B1 in Supplementary Figure 2) containing a single gene (Glyma.01 g048000). The peak SNP is located inside an intron of Glyma.01 g048000 and therefore is unlikely to cause a change in function, whereas all the other significant SNPs are located within the promoter of the gene (within ~ 1 kb of the ATG). Because of the high LD between these markers, these polymorphisms essentially define two haplotypes (alleles), the less-frequent one being associated with improved resistance. Based on the very high degree of association between the peak SNP and this gene, it represents a strong candidate.

## Discussion

### Number of markers

In this study, we used nearly 1.5 M high-quality SNPs. Based on the study of three chromosomal segments, Hyten et al. [14] had estimated that the number of SNPs needed to obtain sufficient coverage (at r^2^ ≥ 0.8) in elite material was somewhere between 9600 and 29,000 markers. More recently, a genome-wide estimation of LD in telomeric and pericentromeric regions led Bastien et al. [3] to conclude that around 60,000 SNPs would be required to achieve extensive coverage. None of the previous GWAM studies investigating SSR resistance achieved such a marker coverage. In two earlier studies using SNP markers, genotyping was conducted with the GoldenGate assay, achieving between 858 [19] and 1142 SNPs [12]. Later GWAM studies used GBS-derived SNP catalogs of 7864 [4], 8397 [15] and 11,811 [30], while another study achieved higher coverage with 25,179 SNPs obtained using SLAF-seq [34]. The highest coverage prior to this work had been achieved recently using the SoySNP50K BeadChip, giving 31,600 and 35,708 SNPs for two AM populations [31]. Here, combining GBS data, WGS data and imputation for missing genotypes, we significantly increased SNP coverage, ensuring for the first time a marker coverage likely conferring exhaustive genome-wide coverage in our association panel. It was previously shown that such imputed data are of high accuracy, with 96.4% of the imputed missing genotypes being in agreement with those obtained at loci in common with the SoySNP50K array [28]. Given the recent increase in availability of WGS data for numerous collections of soybean germplasm, we feel the two-step genotyping approach used in this work will tremendously enhance our ability to perform genome-wide scans with full marker coverage.

### New QTL on Chr01

Within our association mapping panel of 127 Canadian soybean lines, we identified 7 SNPs significantly associated with SSR resistance falling in small segments of only two chromosomes 1 and 15. The first associated region on Chr01, extending over 10 kb, was novel as it did not overlap with any of the previously reported QTLs. In fact, this region didn’t carry any GBS-derived SNPs or those supported by the SoySNP50K BeadChip.

The second QTL was discovered on chromosome 15 and is the exact same marker-trait association reported previously by Bastien et al. [4]. These results were expected given that we exploited essentially the same association panel (except for three lines that were removed) and the same phenotypic data. In this previous work, three other QTLs had been reported (on Chr01, Chr19 and Chr20), but none of them were rediscovered in this work. All three of these associations were characterized by FDR values (ranging between 0.04 and 0.09) that were much higher than the FDR for the QTL on Chr15 (0.01). To investigate possible causes for this apparent lack of reproducibility, we compared the SNP genotypes at all associated SNPs with their corresponding genotypes in our more recent data set resulting from large-scale imputation. Surprisingly, for SNP on chromosome 15, 98% of the genotype calls were identical whereas for the other associated SNPs (on Chr01, Chr19 and Chr20), a much lower level of concordance (60 to 82%) was observed for genotype calls among the lines of the association panel. Based on the work of Torkamaneh and Belzile [26], we believe that the number of SNPs discovered in the work of Bastien et al. [4] proved insufficient to adequately capture haplotypes in this association panel and had led to inaccurate imputation of missing genotypic data.

### QTL validation

Numerous previously reported QTLs for SSR resistance were discovered in different genetic backgrounds, environments, and various sampling populations. Before considering the use of such QTLs for marker-assisted selection, a validation step is highly recommended. The QTL on Chr15 was previously validated by Bastien et al. [4] where resistance alleles reduced lesion length by 12.3 and 17.6 mm in two populations of RILs segregating for associated marker. Such validation has rarely been reported in other studies. Here, we wanted to similarly validate the novel marker-trait association discovered on Chr01 by evaluating SSR partial (or quantitative) resistance (lesion length) in RILs carrying contrasting alleles at this locus. Results showed that lines homozygous for the resistance allele (inherited from Maple Donovan) developed lesions 43 mm shorter than those homozygous for susceptible allele (derived from OAC Bayfield). This phenotypic contrast was highly significant and explained a substantial portion of the phenotypic variation between the parents. Also, the estimated allelic effect at this locus in the segregating RILs was more important than the one measured in the association panel, suggesting that fewer QTLs conditioned SSR resistance in the biparental population than in the association panel. Taken together, these data suggest that the region of Chr01 is associated with SSR resistance.

### QTL detection efficiency and potential uses in genomic selection

The two regions discovered in this work, on Chr01 and Chr15, explained 32 and 15%, respectively, of the phenotypic variation for this trait in our association panel. In previous work, QTLs were reported to explain between 3 to 23% of the variance [4, 15, 21, 30, 31, 34]. Thus, the QTL reported here on chromosome 1 constitutes the most impactful QTL reported for this trait to this date. As the favorable allele was present in a minority (38%) of the elite lines in our panel, there is considerable scope to improve SSR resistance by selecting for this partial resistance QTL in future breeding efforts.

Despite this, it seems that a large proportion of variation for this trait remains uncaptured in our association panel despite the extensive marker coverage. Thus, there could be other undiscovered, potentially impactful QTLs contributing to SSR in this population. In such panels, however, it will always be difficult to capture rare alleles (MAF < 5%) potentially contributing to resistance as the associated markers will have been filtered out. Expanding this work to larger and more diverse panels could help in discovering additional QTLs. Also, the remaining uncaptured heritability may be explained by numerous small-effect QTLs that will be difficult to discover (given the difficulties associated with phenotypic measurements) and would be of limited use in breeding programs as the benefits of a small increase in SSR resistance might not justify the cost of marker-assisted selection for such minor QTLs.

### Candidate genes near the peak SNP on Chr01

When we investigated the associated region on Chr01, we found a single gene in high LD with the peak marker. Except for the peak SNP (located in an intron), all other significantly associated markers were found in the likely promoter of Glyma.01 g048000. Due to the strong association between the peak SNP and this gene, it represents a strong candidate gene. However, due to the lack of annotation allowing one to hypothesize a role for this gene in resistance to Sclerotinia, functional studies will need to be conducted to provide definitive proof of the role of this gene in contributing to resistance. As many of the associated SNPs are in the 5′ upstream region, it would be particularly interesting to study the regulation of this gene.

## Conclusion

We conclude that the knowledge that comes out from this study will promote the addressing of the SSR challenges under sustainable agricultural practices. We believe that this work is another step forward in rendering GWAM data more applicable in plant breeding. We expect that genetic region identified in this study will become a key tool in soybean breeding programs for SSR and enable geneticists and molecular biologists to identify causal resistance genes for SSR in near future.

## Methods

### Association mapping panel

The association mapping panel used for this study was composed of 127 lines taken from a private breeding program (Semences Prograin Inc.) and exhibiting a wide variation in their response to SSR [4]. These were chosen from a larger group of 530 accessions (cultivars/advanced breeding lines) representative of the genetic diversity in Canadian soybean based on previous work [28]. These 127 lines belonged to maturity groups (MGs) ranging from 000 to II except for one line, Williams 82, from MG III. A series of six checks were also included: three cultivars known to offer a good level of SSR partial resistance (Karlo RR, Maple Donovan and S19–90), two moderately resistant cultivars (OAC Bayfield and Williams 82) and one highly susceptible cultivar (Nattosan) [3]. Maple Donovan and Nattosan are commercial cultivars from the Eastern Cereal and Oilseed Research Centre (Agriculture and Agri-Food Canada, Ottawa, Canada), while S19–90 is a commercial cultivar from Syngenta Seeds. Williams 82 was obtained from the American Germplasm Resources Information Network. Seeds of the remaining lines were obtained from Semences Prograin.

### Validation panel

A total of 47 F_6:8_ lines segregating for the candidate QTL region on chromosome 1 (Chr01) were selected to serve as a validation panel. These lines were generated from a cross between the partially resistant Maple Donovan and the susceptible OAC Bayfield.

### Phenotyping

Lines were evaluated for SSR partial resistance using the cotton pad method described in Bastien et al. [3]. For the association panel, the phenotypic data are those previously reported by Bastien et al. [4]. Briefly, plants were sown in a greenhouse in a randomized complete block design with four blocks separated in time (25 Sept, 6 Nov, 8 Dec 2009 and 29 Jan 2010). Experimental units consisted of a total of six plants grown in three 6-L pots (two per pot). The same experimental design was used to characterize the validation panel but with only two blocks separated in time (4 May and 7 Sept 2017) and four plants per experimental unit.

The potting mix was prepared using a mixture of black earth (50%), perlite (30%) and Promix (20%) (Premier Tech Horticulture, Rivière-du-Loup, QC, Canada). At sowing, seeds were inoculated with RhizoStick® inoculant (Becker Underwood, Ames, IA). Plants were grown under a 16-h photoperiod and the day/night temperature was maintained at 26/22 °C.

The inoculum was prepared from strain NB-5 (provided by Dr. S. Rioux of CEROM, Quebec City, QC, Canada) as described in Bastien et al. [3]. Briefly, *S. sclerotiorum* was cultured in potato dextrose broth (PDA) for 3 days until almost reaching saturation. Inoculation was performed once the plants started to flower. First, the suspension was homogenized for 30 s in a blender. Then, pieces (2.7 × 5.5 cm) of cotton pad were soaked in the suspension. The inoculum was applied on the petiole of the lowest node bearing flowers. After inoculation, plants were transferred to a different greenhouse where day/night temperatures were 22 °C/18 °C and high humidity was maintained at 2.5 g/m^3^ with a fogging system. For the validation panel, all plants were inoculated on the same day, while for the association panel, several days were needed because of differences in flowering date. Lesion length was measured 7 d after inoculation.

### SNP genotyping and imputation

The association panel was a part of a larger set of 530 Canadian elite lines on which we had previously performed GBS (*Ape*KI, as per [10]) over time [4, 28]. To maximize data quality and uniformity, all reads (940 M 108-bp single-end Illumina reads) were run on an improved SNP-calling pipeline (Fast-GBS [27];) and on a more recent version of the Williams 82 reference genome (Wm82.a2.v1) [24]. This resulted in a catalogue of 150 K SNPs on which all missing data were imputed using BEAGLE v5 [8] as per Torkamaneh and Belzile [26]. Subsequently, a set of 102 lines (of which 15 were part of the association panel) was subjected to whole-genome sequencing (WGS) [28]. The resulting SNP catalogue (> 4 M SNPs) was used as a reference panel to perform large-scale imputation of missing loci. The SNP data (4.1 M loci) for the 127 lines of the association panel were extracted and filtered using vcftools v0.1.16 [9]. We retained SNPs with a minor allele count (MAC) ≥ 1 and a minor allele frequency (MAF) ≥ 0.05. Linkage disequilibrium (LD) was estimated (using r^2^) for all marker pairs in a sliding window of 50 Kb using PLINK 1.9 [22].

### Analysis of population structure

Given the large size of the SNP catalogue (almost 1.5 M SNPs), pruning was performed using PLINK 1.9 [22] to remove markers in high LD (r^2^ ≥ 0.9). The resulting set of 85 K SNPs was used to assess population structure using fastSTRUCTURE [23] with K set between 1 and 12. The most likely number of subpopulations was estimated using the chooseK tool from fastSTRUCTURE [23].

### Genome-wide association analysis

In view of GWAM, only SNPs having a minor allele frequency (MAF) ≥ 5% in the association panel were used, and this resulted in a catalog of close to 1.5 M filtered SNPs. An association mapping analysis for SSR partial resistance was performed using the phenotypic (mean lesion length) and genotypic data described above with the Genomic Association and Prediction Integrated Tool (GAPIT version 2) [25]. To correct for false-positive associations, a mixed linear model (Q + K model) taking into account both population structure (Q matrix) and relative kinship (K matrix) was used. The Q matrix (for K = 6) was derived from fastSTRUCTURE while the K matrix was generated in TASSEL. Marker-trait associations were deemed significant when the measured *p*-values were below a critical *p*-value corresponding to a false discovery rate (FDR) of 0.1 [5].

### QTL validation

A codominant cleaved amplified polymorphic sequence (CAPS) marker was designed to genotype one of the candidate SNPs (Chromosome 1: 5594765) residing in the haplotype block containing the peak SNP on Chr01. Two specific primers (5′-GTTGTATGGAAGTGCAACTAAAGTTCT-3′ and 5′- GGTACTTTTTCTTACCTTAC GATGA-3′) were used to amplify an 800-bp region encompassing the targeted SNP. The two alleles can be distinguished by digesting the resulting amplicon with *Nmu*CI. The PCR product derived from the allele associated with partial resistance to SSR (present in Maple Donovan) will be cut once while the product obtained after amplification of the allele from the susceptible parent (OAC Bayfield) is not cut. All 47 F_6:8_ lines of the validation panel (described above), along with the two parental lines were genotyped using this CAPS marker.

### Genomic landscape around the peak SNP

LD values from PLINK [22] were extracted for a 2 Mb window around the most significant associated SNP and LD blocks were visualized using Haploview (V4.2) [2] based on r^2^ values. Information about the genes found in the LD block containing the peak association were obtained from SoyBase (www.soybase.org). Functional annotation of nucleotide variation in the region was explored using SnpEFF [35].

## Supplementary information

**Additional file 1: Table S1.** Responses of 127 soybean lines to SSR, 7 days after inoculation. (*) Indicates lines used for WGS (Supplementary file “Supplementary table S1”)

**Additional file 2: Figure S1.** Lesion length distribution across the 127 lines according to alleles at the peak marker on Chr15

**Additional file 3: Figure S2**. LD block plot for the region on Chr01. The arrow shows the position of the significantly associated SNPs. Abbreviations. GBS: Genotyping-by-sequencing. GWAM: Genome-wide association mapping. LD: Linkage disequilibrium. PDA: Potato dextrose agar. PCR: Polymerase chain reaction. QTL: Quantitative trait loci. RILs: Recombinant inbred lines. SLAF-seq: Specific-locus amplified fragment sequencing. SNP: Single nucleotide polymorphism. SSR: Sclerotinia stem rot. USDA: United states department of agriculture. WGS: whole genome sequencing

## Data Availability

A complete catalogue of SNPs in VCF format is publicly available in FigShare (https://figshare.com/s/20a340d7b7cfe26396c9) and all raw sequencing data are publicly available in NCBI Sequence Read Archive (SRA) with the SRP# Study accession, SRP059747 (GBS) and SRP094720 (WGS).
