# Supplementary figures and images for: Genome-wide association mapping of Sclerotinia sclerotiorum resistance in soybean using whole-genome resequencing data
Source: BMC Plant Biol. 2020 May 7;20:195. doi: 10.1186/s12870-020-02401-8 (PMC7333386; doi:10.1186/s12870-020-02401-8)

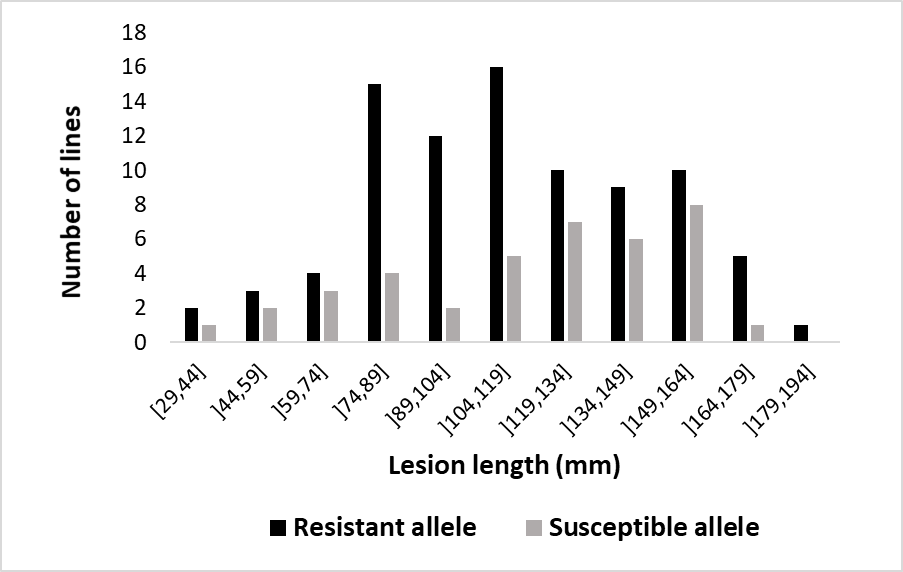

Supplement: Supplementary file 2 — Additional file 2: Figure S1. Lesion length distribution across the 127 lines according to alleles at the peak marker on Chr15 [file 12870_2020_2401_MOESM2_ESM.png]

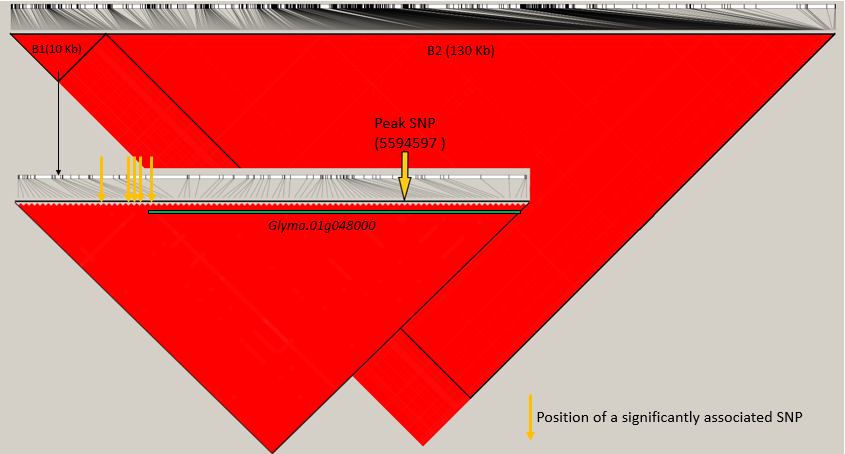

Supplement: Supplementary file 3 — Additional file 3: Figure S2. LD block plot for the region on Chr01. The arrow shows the position of the significantly associated SNPs. Abbreviations. GBS: Genotyping-by-sequencing. GWAM: Genome-wide association mapping. LD: Linkage disequilibrium. PDA: Potato dextrose agar. PCR: Polymerase chain reaction. QTL: Quantitative trait loci. RILs: Recombinant inbred lines. SLAF-seq: Specific-locus amplified fragment sequencing. SNP: Single nucleotide polymorphism. SSR: Sclerotinia stem rot. USDA: United states department of agriculture. WGS: whole genome sequencing [file 12870_2020_2401_MOESM3_ESM.png]
